# Exploring mental health literacy and formal and informal help-seeking intentions among Palestinian adolescents in Israel: insights from the MHLS-Arabic-adolescents tool

**DOI:** 10.3389/fpsyt.2024.1415051

**Published:** 2024-11-20

**Authors:** Fareeda Abo-Rass

**Affiliations:** Takemi Program in International Health, Harvard T.H. Chan School of Public Health, Boston, MA, United States

**Keywords:** adolescents, mental health literacy, formal help-seeking, informal help-seeking minorities, MHLS-Arabic-adolescents, Palestinians, Arabs

## Abstract

**Background:**

Mental health literacy (MHL) is a crucial concept in mental health because it promotes positive mental health outcomes and behaviors across various demographics, including adolescents.

**Aims:**

This study utilized the MHLS-Arabic-Adolescents tool to investigate MHL among Palestinian adolescents in Israel, aiming to explore its significance in determining intentions to seek formal and informal mental health help separately.

**Methods:**

A cohort of 172 adolescents (with a mean age of 16.25 ± 1.25 years, comprising 64% females) completed assessments measuring their intention for both formal and informal help-seeking, levels of psychological distress, MHL, and various sociodemographic and clinical characteristics. This study uses the same dataset from previous research on formal help-seeking, with an expanded focus on informal help-seeking and MHL using an adapted tool.

**Results:**

Participants reported medium to high levels of MHL factors, demonstrating stronger intentions to seek informal help compared to formal. “Knowledge of where to seek information” and “Attitude toward mental illness” emerged as the significant MHL factors determining formal help-seeking intentions, whereas ‘attitudes toward mental illness’ was identified as the sole determinant of intentions to seek informal help.

**Conclusions:**

This study highlights the importance of MHL factors in shaping adolescents’ intentions to seek formal and informal mental health help. It emphasizes the need to strengthen informal support networks, improve adolescents’ attitudes toward mental illness, and facilitate access to mental health information. These insights offer valuable guidance for intervention programs aimed at promoting both formal and informal help-seeking behaviors among Palestinian Arab adolescents in Israel and similar groups.

## Introduction

Mental health literacy (MHL) has emerged as a pivotal concept in the mental health domain since its inception, with numerous studies underscoring its role in fostering positive mental health outcomes and behaviors (1–3). These include intentions and behaviors related to seeking help and utilizing mental health services (4, 5).

According to Jorm and colleagues’ definition of MHL (1997), it encompasses knowledge and beliefs about mental disorders that facilitate their recognition, management, or prevention. This concept comprises six dimensions: Ability to recognize disorders; Knowledge of risk factors and causes; Knowledge of self-treatment; Knowledge of professional help available, Knowledge of where to seek information; and Attitudes that promote recognition or appropriate help-seeking behavior (6). The Mental Health Literacy Scale (MHLS) developed by O’Connor and Casey (7) is the primary instrument for assessing MHL. Comprising 35 items, the MHLS evaluates mental health knowledge, attitudes, and behaviors across the six dimensions outlined in Jorm’s definition. This tool has been utilized to evaluate MHL among diverse ethnic groups and populations worldwide, showcasing its adaptability and underscoring its crucial role in propelling MHL research forward (8). Recently, the MHLS tool underwent translation into Arabic and was validated and adopted for adolescents aged 14-18 (Author). This validation process resulted in the creation of the MHLS-Arabic-Adolescents tool, consisting of 31 items organized into 5 factors: Recognition of Anxiety and Depression Disorders; Mental Health Knowledge; Knowledge of where to seek information; Attitude toward mental illness; and Attitudes towards people with a mental health problem.

The Palestinian citizens of Israel are Arabic speakers who belong to the Arab world and culture, constituting the largest ethnic minority in Israel, comprising 21.1% of the population, while Israeli Jews form the majority (9; CBS). Around 40% of Palestinian minors are under 18, totaling approximately 200,000 adolescents aged 14-18. Notably, 45.3% of them live below the poverty line (9). Research, such as the Israel Survey of Mental Health among Adolescents, suggests Palestinians experience higher rates of mental health problems (10, 11). According to the Galilee Study, prevalence rates of mental health disorders among Palestinian adolescents stand at 19.2% for any disorder, 15.8% for internalizing disorders, and 4.2% for externalizing disorders (11). Disparities in mental health service utilization are apparent, with only 19.8% of Palestinian adolescents accessing services compared to 49.9% of Jewish adolescents (12). Overall, research on mental health among Palestinian adolescents in Israel, particularly concerning MHL and seeking formal and informal mental health help, is severely lacking. The only existing study on MHL among this population, conducted by the author and colleagues (4), examines various determinants of intentions to seek formal mental health help among Palestinian adolescents in Israel, including MHL. However, this study utilized the MHLS developed by O’Connor and Casey (7), which is not fully adapted to adolescents or Arab populations. Additionally, the study focused solely on formal help-seeking, leaving a gap in understanding intentions to seek informal help.

Hence, this brief report aims to utilize the MHLS-Arabic-Adolescents tool to explore MHL among Palestinian Arab adolescents who are citizens of Israel, examining its role as a determinant of intentions to seek both formal and informal mental health help separately. Although this study relies on the same dataset as the previously mentioned study on Palestinian adolescents in Israel, it marks the first use of the MHLS-Arabic-Adolescents tool in such research.

## Methods

### Sample and procedure

This research engaged 172 adolescents who met specific criteria: they were Palestinian-Arab citizens of Israel aged between 14 and 18. Research assistants utilized various social media parent groups to promote the study and recruit participants. The promotional materials disseminated details about the study and contact information for the researchers and invited interested parents to reach out to the principal researcher (author).

Upon contacting the author and providing consent for their children’s participation in the study, parents received a link containing information about the study and the questionnaire. They were requested to provide informed consent for their children’s involvement and were guided to share the questionnaire directly with their children. The children were instructed to complete the questionnaire independently.

Before completing these questionnaires, the adolescent participants indicated their agreement to participate in the study by clicking the assent button on the questionnaire. Additionally, they confirmed their parents’ consent and verified that they had received their survey link. The link explained the study and provided information about the researcher’s commitment to maintaining confidentiality and anonymity, voluntary participation, and withdrawal at any stage. The questionnaire administered to the adolescents comprised several sections. It commenced with explaining the study’s objectives and providing contact details for the researcher. Subsequently, participants completed a socio-demographic questionnaire, followed by an intention for Formal Help-Seeking and Informal Help-Seeking questionnaire, a psychological distress questionnaire, and a mental health literacy questionnaire.

The research protocol was approved by the Ethics Committee of Zefat Academic College approved the research protocol (approval number 16-2023).

### Measures

#### Mental health literacy

The MHLS-Arabic-Adolescents was employed to evaluate MHL (author). This self-report measure includes 31 items across five factors: (1) Recognition of Anxiety and Depression Disorders (three items, an example item, *‘If someone experienced a low mood for two or more weeks, had a loss of pleasure or interest in their normal activities and experienced changes in their appetite and sleep then to what extent do you think it is likely they have Major Depressive Disorder’*); (2) Mental Health Knowledge (nine items, an example item, *‘To what extent do you think it would be helpful for someone to improve their quality of sleep if they were having difficulties managing their emotions’*); (3) Knowledge of where to seek information (four items; an example item, *‘I am confident I have access to resources (e.g., GP, internet, friends) that I can use to seek information about mental illness’*); (4) Attitude toward mental illness (six items, an example item, ‘A mental illness is not a real medical illness’); and (5) Attitudes towards people with a mental health problem (nine items, an example item, How willing would you be to employ someone if you knew they had a mental illness’).

All items of Factor (1) were assessed using a four-point Likert scale, ranging from 1 (very unlikely) to 4 (very likely). Items related to Factor (2) were rated on a four-point Likert scale, varying from 1 (very unlikely) to 4 (very likely) or from 1 (very unhelpful) to 4 (very helpful). Factors (3), (4), and (5) were evaluated using a 5-point Likert scale, spanning from 1 (strongly disagree) to 5 (strongly agree). Scores for items within each factor were summed, with higher scores indicating greater literacy in that particular aspect of MHL (for example, a higher score on the factor ‘Recognition of Anxiety and Depression Disorders’ means higher knowledge of and higher ability to recognize Anxiety and Depression. A higher score on the factor ‘Attitudes towards people with a mental health problem’ means a more positive attitude toward people with mental disorders). High internal reliability was observed for all five factors of the MHLS-Arabic-Adolescents, with Cronbach’s alpha values ranging from α = 0.72 to 0.87.

#### Intention for formal help-seeking and informal help-seeking

The General Help-Seeking Questionnaire (GHSQ; 13) was employed to assess participants’ inclination toward seeking formal and informal assistance. This questionnaire prompted individuals to rate their likelihood of seeking help from various formal and informal sources when encountering personal or emotional issues. Formal sources encompassed mental health professionals, teachers, phone helplines, and doctors/GPs, while informal sources included boyfriend/girlfriend, parents, relatives, religious leaders, and other individuals (such as colleagues, neighbors, friends, etc.). Participants rated their likelihood of seeking help from each source on a scale of 1 to 7, where 1 indicated “extremely unlikely” and 7 indicated “extremely likely.” Two indices were calculated by averaging scores across all items in each category (formal and informal), with higher scores indicating a greater propensity to seek help from formal or informal sources. The questionnaire underwent translation from English to Arabic using a back-and-forth method for this study. It exhibited moderate internal reliability, with Cronbach’s α values of.68 and 0.52 for formal and informal sources, respectively.

#### Psychological distress

The General Health Questionnaire (GHQ-12; 14) was utilized to evaluate psychological distress. Consisting of 12 items, respondents rated their experiences on a 4-point Likert scale. Reverse scoring was applied to seven items. Questions inquired about recent feelings across various domains, such as decision-making capabilities and enjoyment of daily activities. Total scores were derived by summing all items where a scoring range of 0, 0, 1, and 1 was assigned instead of 1, 2, 3, and 4. Scores ranged from 0 to 12, with higher scores indicating elevated levels of psychological distress. The questionnaire’s validation in Arabic has been established previously (15), and in our study, we observed a high internal reliability of the scale (α = 0.79).

#### Sociodemographic and clinical characteristics

The sociodemographic and clinical questionnaire included questions regarding age, gender, residence area (North, Central, South), subjective socioeconomic status (rated on a scale from 1 to 10, where 1 represents the lowest and 10 represents the highest assessment of one’s and their family’s socio-economic situation), and Hebrew proficiency (rated as Don’t know/poor, average, excellent). Additionally, participants indicated whether they had received a current or past diagnosis of mental illness (Yes, No) or had engaged in mental health treatment (Yes, No).

#### Statistical analysis

Data were coded and analyzed using SPSS-25. Descriptive statistics were employed to illustrate participants’ characteristics and main variables. Pearson correlations and t-tests assessed associations between sociodemographic variables, Mental Health Literacy (MHL) factors, and intentions for formal and informal help-seeking. Two hierarchical multiple regression analyses were conducted to examine determinants of intentions for formal help-seeking and informal help-seeking.

## Results

### Sample description

**Table 1** presents the sociodemographic and clinical characteristics of the participants. The mean age of the participants was 16 years (SD=1.25). Most participants were female (61.8%) and resided in the Central area of Israel (51.7%). Moreover, a significant proportion of participants reported no prior diagnosis of mental illness (93%) or history of mental health treatment (87.1%). On average, participants reported a psychological distress score of 3.59 (SD=1.23; Range 0-12). Approximately half of the participants had an average level of proficiency in Hebrew (48.8%) and reported moderate to high subjective socioeconomic status, with a mean score of 6.56 (SD=1.88; Range= 1-10).

**Table 1:** Participants` sociodemographic and clinical characteristics.

	Entire sample (*N* = 172)
**Age** *M* (*SD*)	16.25 (1.25)
Gender n (%)	
Male	62 (36)
Female	110 (64)
Residence Area n (%)	
North	69 (40.4)
Central	89 (51.7)
South	14 (7.9)
**Subjective Socioeconomic Status** M (SD)	6.56 (1.88)
Hebrew Proficiency n (%)	
Don’t know/Poor	64 (37.2)
Average	84 (48.8)
Excellent	24 (14)
Mental Illness n (%)	
Yes	12 (7)
No	160 (93)
Mental health Treatment n (%)	
Yes	21 (12.9)
No	151 (87.1)
**Psychological Distress** M (SD)	3.59 (1.25)

### Levels of mental health literacy, intention for formal help-seeking, and intention for informal help-seeking

**Table 2** presents the mean, standard deviation, and possible range of MHL factors, Intention for Formal Help-Seeking, and Intention for Informal Help-Seeking. Participants reported medium or high levels in all MHL factors, with a slightly lower-than-average intention to seek formal help but slightly higher-than-average intention to seek informal help.

**Table 2:** Possible ranges, means, and standard deviations of the study’s variables.

	Possible Range	*M (SD)*
Intention for Formal Help-Seeking	1-7	2.88 (1.32)
Intention for Informal Help-Seeking	1-7	3.58 (0.94)
Factor 1: Recognition of Anxiety and Depression Disorders	3-12	8.39 (2.46)
Factor 2: Mental Health Knowledge	9-36	22.26 (6.87)
Factor 3: Knowledge of where to seek information	4-20	12.69 (3.87)
Factor 4: Attitude toward mental illness	6-30	23.01 (4.56)
Factor 5: Attitudes towards people with a mental health problem	6-45	29.95 (6.61)

### MHL as determinants of intentions to for formal help-seeking, and intention for informal help-seeking

The hierarchical multiple regression analysis results in **Table 3** elucidate the predictors of both formal and informal help-seeking intentions. For formal help-seeking, the first step involved integrating sociodemographic and clinical variables identified as significantly associated with formal help-seeking at the bivariate level as control variables. These variables comprised subjective socioeconomic status (r = 0.24, p <.001), psychological distress (r = −0.24, p <.001), and mental illness diagnosis (t_(169)_ = 2.43, p <.05). This initial set of factors collectively accounted for 9% of the variance in formal help-seeking. Subsequently, all MHL factors significantly correlated with formal help-seeking were included in the second step. These encompassed factors 3, Knowledge of where to seek information (r = 0.39, p <.001), and factor 4, Attitude toward mental illness (r = 0.33, p <.001). Upon inclusion of these MHL dimensions, the Cox R2 increased to 0.24, signifying that the model explained 24% of the variation in formal help-seeking intention. The full regression model yielded significance (F _(5,160)_ = 11.16, p < 0.001), underscoring that higher socioeconomic status (β = 0.17, p < 0.05), decreased psychological distress (β =-0.17, p < 0.05), better Knowledge of where to seek information (β = 0.29, p < 0.001), and more favorable Attitude toward mental illness (β = 0.27, p < 0.001) were the main determinants of formal help-seeking intention.

**Table 3:** Regression analysis for intention for formal help-seeking and informal help-seeking (N = 172).

		Formal help-seeking				Informal help-seeking	
		β	Δ adj. *R* ^2^			β	Δ adj. *R* ^2^
**Step 1**			.09***	**Step 1**			0.14***
	Subjective socioeconomic status	0.17*			Gender	-0.23**	
	Psychological distress	-0.17*			Subjective socioeconomic status	0.14	
	Mental illness diagnosis	-0.12			Psychological distress	-0.16*	
					Mental illness diagnosis	-0.14	
**Step 2**			0.15***	**Step 2**			0.04***
	Knowledge of where to seek information	0.29***			Knowledge of where to seek information	0.14	
	Attitude toward mental illness	0.27***			Attitude toward mental illness	0.16*	
**Total Adj. R2**			0.24	**Total Adj. R2**			0.18

*p<.05, **p<.01, ***p<.001.

For informal help-seeking, the initial step involved incorporating sociodemographic and clinical variables identified as significantly associated with formal help-seeking at the bivariate level as control variables, including gender (t_(170)_ = 3.42, p <.001), subjective socioeconomic status (r = 0.20, p <.01), psychological distress (r = −0.28, p <.01), and mental illness diagnosis (t_(170)_ = 2.91, p <.01). It’s notable that sociodemographic and clinical characteristics accounted for 14% of the variance in informal help-seeking. In the second step, all MHL dimensions found to be significantly related to informal help-seeking were included. These encompassed factor 3, Knowledge of where to seek information (r = 0.23, p <.001), and factor 4, Attitude toward mental illness (r = 0.18, p <.05). Upon adding these significant MHL dimensions, the Cox R2 increased to 0.18, indicating that the estimated model explained 18% of the variation in intention for informal help-seeking. The full regression model demonstrated significance (F _(6,160)_ = 7.02, p <.001), underscoring that being male (β = -0.23, p < 0.01), decreased psychological distress (β = -0.16, p < 0.05), and more favorable Attitude toward mental illness (β = 0.16, p < 0.05) were the main determinants of intention for informal help-seeking.

## Discussion

This study delved into MHL using the MHLS-Arabic-Adolescents tool, exploring its various factors’ levels and their significance as determinants of intention for formal help-seeking and informal help-seeking among Palestinian adolescents in Israel. The findings illuminate several noteworthy aspects.

Regarding MHL, the research findings indicate that Palestinian Adolescents in Israel exhibit relatively high levels in the MHL factors of Recognition of Anxiety and Depression Disorders, Attitudes toward mental illness, and Attitudes toward people with mental health problems. While they demonstrate average levels of mental health knowledge and knowledge of where to seek information. This finding was quite unexpected, given that a recent literature review indicates low MHL among adolescents from low- and middle-income countries and marginalized groups (16). However, we cannot fully compare the results because of the different tools used to assess MHL. Generally, the elevated to high levels of MHL across all factors can be attributed to the high socioeconomic status prevalent in the sample, as well as the fact that more than half of the participants are female (17, 18). Comparing the findings of this study to the previous one that used the unadjusted MHLS tool (4), the previous results also indicated average or above-average levels on all MHL dimensions. However, the contribution of this new report lies in the more detailed factorization enabled by the adapted tool. This adjustment allowed for a more apparent distinction between MHL dimensions, providing deeper insights into specific factors such as Recognition of Anxiety and Depression Disorders and Attitudes toward people with mental health problems. Moreover, this also allows for a deeper understanding of how these factors specifically relate to formal and informal help-seeking intentions.

The high levels of MHL factors related to anxiety and depression disorders reported by the participants suggest a promising trend in terms of awareness and recognition of these prevalent mental health conditions, considering that anxiety and depression are the most common mental disorders experienced by adolescents globally (19). These high levels may reflect the impact of socio-political factors on mental health within this population, as they face unique challenges, including discrimination, political tensions, and socio-economic disparities (20). Such stressors can contribute to heightened rates of anxiety and depression among this population, prompting individuals to develop a deeper understanding of these conditions as a means of coping (21). Contrarily, the revelation that participants reported only an average level, rather than a high level, of knowledge regarding where to seek information related to mental health demands particular attention, particularly given their adolescent status. This finding underscores existing gaps in access to resources and limitations in awareness campaigns targeting this age group (12). Also, levels of knowledge regarding mental health were found to be moderate among the study sample, underscoring the necessity for policymakers and professionals to prioritize efforts aimed at enhancing awareness of mental health and making the pathways to seek information and help accessible and available among Palestinian adolescents in Israel.

Furthermore, the results indicate that participants reported a greater intention to seek informal help compared to formal help. Subsequent analyses of this data highlight a statistically significant difference between the two help-seeking paths. These findings align with previous research demonstrating a preference for informal care among various adolescent populations, including Asian and Caucasian adolescents in the US, those in South India, and Chilean adolescents (22–24). The observed preference among Palestinian adolescents in Israel echoes broader societal trends within the Palestinian community in Israel, underscoring the traditional and collective nature of their society and culture. This collective orientation emphasizes family cohesion and community support, favoring traditional help-seeking methods over Western therapy. Mental health challenges are often addressed through a community-centered approach, with family and trusted figures providing help and support (25). In such contexts, Palestinians often rely on their community for support in times of need (21, 26). This finding underscores the importance of understanding adolescents’ needs and preferences. The study cautions against assuming that formal support systems are their default or preferred choice and advises against pressuring them towards such options against their inclinations. Instead, in line with this finding, it might be better to concentrate on empowering their informal support networks and equipping them with MHL. By enhancing the knowledge and awareness of these informal sources, including parents and peers, these sources can offer effective support to adolescents.

In the context of MHL as a determinant of intentions for formal and informal mental health help-seeking, the study revealed that the factor ‘knowledge of where to seek information’ is a significant determinant of the intention to seek formal help but not informal help. This underscores the importance of information access in facilitating access to formal support, emphasizing the need for initiatives focused on improving access to mental health-related information, especially among this disadvantaged demographic. Such endeavors are vital, considering the role of information accessibility in offering mental health assistance and addressing gaps in mental health care (2). Secondly, this finding may suggest that informal help is readily available and more easily accessible without the need to seek specific information. This observation further emphasizes the importance of strengthening these informal support networks. This conclusion is further supported by the finding that socioeconomic status emerges as a statistically significant determinant of formal help-seeking but not informal. Adolescents from higher socioeconomic backgrounds are more inclined to seek formal help, whereas socioeconomic status does not appear to affect their ability to seek informal help.

Simultaneously, the “attitudes toward mental illness” factor of MHL emerged as a statistically significant determinant of both informal and formal help-seeking. This aligns with findings from studies conducted among the Palestinian minority in Israel as well as among adolescents from other groups worldwide (4, 27, 28). What sets this study apart is its revelation that attitudes towards mental illness, rather than attitudes towards people with mental disorders, are the significant and determining factors. This underscores a distinction between the two concepts, which are typically presented as a single factor in the original Mental Health Literacy Scale. It also emphasizes the importance of focusing on improving attitudes towards mental health and mental disorders specifically to encourage help-seeking, whether formal or informal.

Lastly, this study has several limitations. Firstly, a culturally homogeneous sample may limit the generalizability of findings. Secondly, convenience sampling introduces selection bias, potentially limiting applicability. Additionally, participants’ characteristics may contribute to their high interest in and knowledge of mental health issues. Online data collection may also favor frequent Internet users. The data primarily rely on self-report measures, which may lead to potentially inaccurate interpretations of certain questions. This issue is compounded by the absence of a researcher to make necessary corrections. This is particularly significant in the context of mental health-related issues. Finally, the observational, cross-sectional design prevents establishing causal relationships.

Future studies can address these limitations by including a more culturally diverse sample, using random sampling methods, and considering participants with varying levels of interest in mental health. Employing mixed data collection methods, including offline approaches, can mitigate the bias towards frequent Internet users. Furthermore, adopting a longitudinal design can help establish causal relationships.

## Conclusions and implications

In contrast to the previous paper that relied on the MHLS, this study sheds light on MHL among Palestinian Arab adolescents in Israel, utilizing the MHLS-Arabic-Adolescents tool to explore its significance in determining intentions for formal and informal help-seeking. This tool provided a more culturally and developmentally appropriate framework, allowing for a more precise exploration of MHL’s significance in determining formal and informal help-seeking intentions. The research demonstrates varying literacy levels across different MHL factors, highlighting the need to prioritize enhancements in the “Knowledge of where to seek information” and “Mental Health Knowledge.”

The findings enrich the current understanding of factors influencing adolescents’ propensity to seek both formal and informal assistance. This holds particular relevance for adolescents from ethnic minority backgrounds, emphasizing the pivotal role of MHL factors in shaping these intentions. Specifically, knowledge of where to seek information and attitudes toward mental illness emerged as significant determinants of the intention for formal help-seeking, while attitudes toward mental illness were the sole significant MHL factors influencing intention for informal help-seeking. These insights are instrumental for designing intervention programs that foster help-seeking behaviors, whether formal or informal. Moreover, our findings highlight a distinct preference among Palestinian adolescents for seeking informal help over formal help, echoing trends observed in adolescent populations. This underscores the need to acknowledge adolescents’ preferences and strengthen their informal support networks to provide effective support.

Despite the importance of this research and the use of a valid tool for Arabic, particularly among the adolescent age group, this tool is still based on a Western framework. Palestinian adolescents are not only an ethnic minority but also a group that lives under continuous political conflict and war. This is true for many ethnic minorities worldwide living in war zones. Mental health literacy as a concept is still not fully understood among groups living under such conditions, especially adolescents. Therefore, this concept requires further development to clearly define what mental health literacy means for people living under these circumstances.

## Data Availability

The raw data supporting the conclusions of this article will be made available by the authors, without undue reservation.

## References

[1] Jafari A Nejatian M Momeniyan V Barsalani FR Tehrani H. Mental health literacy and quality of life in Iran: a cross-sectional study. BMC Psychiatry. (2021) 21:1–11. doi: 10.1186/s12888-021-03507-5 34641793 PMC8507341

[2] Jorm AF. Mental health literacy: Empowering the community to take action for better mental health. Am Psychol. (2012) 67:231–43. doi: 10.1037/a0025957 22040221

[3] Olyani S Gholian Aval M Tehrani H Mahdiadeh M. School-based mental health literacy educational interventions in adolescents: a systematic review. J Health Literacy. (2021) 6:69–77.

[4] Abo-Rass F Nakash O Gelaye B Khatib A AboJabel H. Determinants of intentions to seek formal mental health help among Palestinian adolescents in Israel. Int J Soc Psychiatry. (2024) 70(4):720–9. doi: 10.1177/00207640231224658 38312061

[5] Noroozi A Khademolhosseini F Lari H Tahmasebi R. The mediator role of mental health literacy in the relationship between demographic variables and health-promoting behaviours. Iranian J Psychiatry Behav Sci. (2018) 12(2):e12603. doi: 10.5812/ijpbs.12603

[6] Jorm AF. Mental health literacy: Public knowledge and beliefs about mental disorders. Br J Psychiatry. (2000) 177:396–401. doi: 10.1192/bjp.177.5.396 11059991

[7] O’Connor M Casey L. The mental health literacy scale (MHLS): A new scale-based measure of mental health literacy. Psychiatry Reasrerch. (2015) 229:511–6. doi: 10.1016/j.psychres.2015.05.064 26228163

[8] Wei Y Kutcher S. Innovations in practice: ‘Go-to’ educator training on the mental health competencies of edu- cators in the secondary school setting: A program evalua- tion. Child and Adolescent Mental Health. (2014) 19(3):219–222. doi: 10.1111/camh.12056 32878378

[9] Central Bureau of Statistics. Population in Israel by Population Group, Religion, Sex, and Age, End of 2022 (2023). Available online at: https://www.cbs.gov.il/he/subjects/Pages/%D7%90%D7%95%D7%9B%D7%9C%D7%95%D7%A1%D7%99%D7%99%D7%94.aspx (Accessed 1 April 2024).

[10] Farbstein I Mansbach-Kleinfeld I Levinson D Goodman R Levav I Vograft I. Prevalence and correlates of mental disorders in Israeli adolescents: results from a national mental health survey. J Child Psychol Psychiatry. (2010) 51:630–9. doi: 10.1111/j.1469-7610.2009.02188.x 19874426

[11] Daeem R Mansbach-Kleinfeld I Farbstein I Goodman R Elias R Ifrah A. Correlates of mental disorders among minority Arab adolescents in Israel: results from the Galilee Study. Israel J Health Policy Res. (2019) 8:14–4. doi: 10.1186/s13584-018-0281-5 PMC6340179 30665458

[12] Mansbach-Kleinfeld I Farbstein I Saragusti I Karmon G Apter A Ifrah A. (2013). Mapping of mental health clinics for children and adolescents in Israel: Geographic and structural disparities, in: Paper presented at the 5th International Jerusalem Conference of Health Policy.

[13] Wilson CJ Deane FP Ciarrochi J Rickwood D. Measuring help-seeking intentions: Properties of the gen- eral help-seeking questionnaire. Canadian Journal of Counselling. (2005) 39(1):15.

[14] Goldberg DP. The detection of psychiatric illness by questionnaire: A technique for the identification and assess- ment of non-psychotic psychiatric illness. Oxford University Press. (1972).

[15] Daradkeh T Ghubash R El-Rufaie O. Reliability, validity, and factor structure of the Arabic version of the 12-item general health questionnaire. Psychological Reports. (2001) 89(1):85–94. doi: 10.2466/PR0.89.5.85-94 11729557

[16] Renwick L Pedley R Johnson I Bell V Lovell K Bee P. Mental health literacy in children and adolescents in low- and middle-income countries: A mixed studies systematic review and narrative synthesis. Eur Child Adolesc Psychiatry. (2022). doi: 10.1007/s00787-022-01997-6 PMC11032284 35570227

[17] Attygalle UR Perera H Jayamanne BDW. Mental health literacy in adolescents: ability to recognise problems, helpful interventions and outcomes. Child Adolesc Psychiatry Ment Health. (2017) 11(1):38. doi: 10.1186/s13034-017-0176-1 28814972 PMC5557470

[18] Bjørnsen HN Espnes GA Eilertsen M-EB Ringdal R Moksnes UK. The relationship between positive mental health literacy and mental well-being among adolescents: implications for school health services. J School Nurs. (2019) 35(2):107–16. doi: 10.1177/1059840517732125 PMC7323733 28950750

[19] World Health Organization. Mental Health of Adolescents (2021). Available online at: https://www.who.int/activities/improving-health-literacy (Accessed April 8, 2024).

[20] Mansbach-Kleinfeld U Daeem R. Mental Health Status, Service Use, and Help-Seeking Practices of Children and Adolescents among Palestinian Citizens in Israel. In: Haj-Yahia M Nakash O Levav I, editors. Mental Health and Palestinian Citizens in Israel. Indiana University Press (2019). p. 176–97.

[21] Abu-Kaf S. Mental Health Issues among Palestinian Women in Israel. In: Haj-Yahia M Nakash O Levav I, editors. Mental Health and Palestinian Citizens in Israel. Indiana University Press (2019). p. 165–76.

[22] Chiang S Chin CA Meyer EW Sust S Chu J. Asian american adolescent help-seeking pathways for psychological distress. Asian Am J Psychol. (2022) 13:194–206. doi: 10.1037/aap0000241

[23] Ogorchukwu JM Sekaran VC Nair S. Mental health literacy among late adolescents in south India: what they know and what attitudes drive them. Indian J psychol Med. (2016) 38:234–41. doi: 10.4103/0253-7176.183092 PMC4904760 27335519

[24] Olivari C Mellado CLA Casañas R Espinosa-Díaz N Fuster-Villaseca J. Preferred sources of help for mental health problems among Chilean adolescents: a descriptive study. Boletín Médico Del Hosp Infantil México. (2021) 78. doi: 10.24875/bmhim.20000182 33939686

[25] Haj-Yahia M. The Palestinian Family in Israel: Its Collectivist Nature, Structure, and Implications for Mental Health Interventions. In: Haj-Yahia M Nakash O Levav I, editors. Mental Health and Palestinian Citizens in Israel. Indiana University Press (2019). p. 165–76.

[26] Al-Krenawi A. Attitudes, Beliefs, and Stigma Toward Mental Health Issues among Palestinian Citizens in Israel. In: Haj-Yahia M Nakash O Levav I, editors. Mental Health and Palestinian Citizens in Israel. Indiana University Press (2019). p. 165–76.

[27] Aguirre Velasco A Cruz ISS Billings J Jimenez M Rowe S. What are the barriers, facilitators and interventions targeting help-seeking behaviours for common mental health problems in adolescents? A systematic review. BMC Psychiatry. (2020) 20:293–3. doi: 10.1186/s12888-020-02659-0 PMC7291482 32527236

[28] Radez J Reardon T Creswell C Lawrence PJ Evdoka-Burton G Waite P. Why do children and adolescents (not) seek and access professional help for their mental health problems? A systematic review of quantitative and qualitative studies. Eur Child Adolesc Psychiatry. (2021) 30:183–211. doi: 10.1007/s00787-019-01469-4 31965309 PMC7932953

